# Notochordal cells protect nucleus pulposus cells from degradation and apoptosis: implications for the mechanisms of intervertebral disc degeneration

**DOI:** 10.1186/ar3548

**Published:** 2011-12-29

**Authors:** W Mark Erwin, Diana Islam, Robert D Inman, Michael G Fehlings, Florence WL Tsui

**Affiliations:** 1 University of Toronto and Toronto Western Hospital, Mc13-310, 399 Bathurst Street, Toronto M5T 2S8; 2 Institute of Medical Science, 2374 Medical Sciences Building, 1 King's College Circle, University of Toronto, Toronto, Ontario, Canada, M5S 1A8; 3 Toronto Western Research Institute, 399 Bathurst Street, Toronto M5T 2S8, Toronto Ontario, Canada; 4 Division of Neurosurgery, Toronto Western Hospital, 399 Bathurst St., WW 4-447, Toronto, ON M5T 2S8; 5 Department of Immunology, Medical Sciences Building, Room 5271, University of Toronto, 1 King's College Circle, Toronto, Ontario M5S 1A8

## Abstract

**Introduction:**

The relative resistance of non-chondrodystrophic (NCD) canines to degenerative disc disease (DDD) may be due to a combination of anabolic and anti-catabolic factors secreted by notochordal cells within the intervertebral disc (IVD) nucleus pulposus (NP). Factors known to induce DDD include interleukin-1 beta (IL-1ß) and/or Fas-Ligand (Fas-L). Therefore we evaluated the ability of notochordal cell conditioned medium (NCCM) to protect NP cells from IL-1ß and IL-1ß +FasL-mediated cell death and degeneration.

**Methods:**

We cultured bovine NP cells with IL-1ß or IL-1ß+FasL under hypoxic serum-free conditions (3.5% O_2_) and treated the cells with either serum-free NCCM or basal medium (Advanced DMEM/F-12). We used flow cytometry to evaluate cell death and real-time (RT-)PCR to determine the gene expression of aggrecan, collagen 2, and link protein, mediators of matrix degradation *ADAMTS-4 *and *MMP3*, the matrix protection molecule *TIMP1*, the cluster of differentiation (*CD)44 *receptor, the inflammatory cytokine *IL-6 *and *Ank*. We then determined the expression of specific apoptotic pathways in bovine NP cells by characterizing the expression of activated caspases-3, -8 and -9 in the presence of IL-1ß+FasL when cultured with NCCM, conditioned medium obtained using bovine NP cells (BCCM), and basal medium all supplemented with 2% FBS.

**Results:**

NCCM inhibits bovine NP cell death and apoptosis via suppression of activated caspase-9 and caspase-3/7. Furthermore, NCCM protects NP cells from the degradative effects of IL-1ß and IL-1ß+Fas-L by up-regulating the expression of anabolic/matrix protective genes (aggrecan, collagen type 2, CD44, link protein and TIMP-1) and down-regulating matrix degrading genes such as MMP-3. Expression of *ADAMTS-4*, which encodes a protein for aggrecan remodeling, is increased. NCCM also protects against IL-1+FasL-mediated down-regulation of *Ank *expression. Furthermore, NP cells treated with NCCM in the presence of IL-1ß+Fas-L down-regulate the expression of *IL-6 *by almost 50%. BCCM does not mediate cell death/apoptosis in target bovine NP cells.

**Conclusions:**

Notochordal cell-secreted factors suppress NP cell death by inhibition of activated caspase-9 and -3/7 activity and by up-regulating genes contributing anabolic activity and matrix protection of the IVD NP. Harnessing the restorative powers of the notochordal cell could lead to novel cellular and molecular strategies in the treatment of DDD.

## Introduction

Degenerative disc disease (DDD) is an extremely common and costly healthcare condition for which there is no curative strategy [1]. Given the lack of a biological strategy for regeneration of the degenerating disc, a therapeutic intervention that may offer restorative qualities to the disc is a much needed and widely sought goal. The ideal biological agent might reactivate homeostatic mechanisms innately inherent to the healthy intervertebral disc (IVD). The capacity to re-establish equilibrium between catabolic and anabolic tissue remodeling would represent the ideal regenerative strategy for the treatment of DDD.

With respect to potential biological therapies, lessons learned from the study of the non-chondrodystrophic canine (NCD canine) IVD might provide essential molecular clues for restoration of homeostasis to the disc. The NCD canine is unique among the canine sub-species in that this animal is relatively resistant to the development of DDD. Notably, NCD canines preserve their notochordal cell populations throughout life [2,3]. Thus, there is an emerging body of evidence indicating that notochordal cells confer anabolic capacity upon NP cells and that their absence is associated with susceptibility to degenerative changes [2,4,5].

### Apoptosis plays a central role in DDD development

Regulation of cellular turnover is vital to tissue homeostasis. Apoptosis is a highly regulated form of programmed cell death that classically involves two main pathways, the intrinsic (mitochondrial-dependent) and extrinsic (death receptor or Fas-dependent) pathways. It has been established that some cells, classified as Type I cells, function independently of the mitochondria and signal via Fas-induced apoptotic cell death involving the caspase-8 pathway. Other cells have a critical reliance upon the mitochondria whereby apoptosis is mediated via caspase-9 and are known as Type II cells [6,7]. The initial explorations of these pathways involved the use of knock-out mice leading to the conclusions that some tissues are primed to respond to apoptotic stimuli in a Type I versus Type II manner [7,8].

The classic extrinsic (CD95/Fas receptor) apoptotic pathway is activated by soluble Fas ligand (Fas-L) binding to the CD95 or Fas receptor that in turn activates caspase-8 followed by sequential activation of executioner caspases-7 and -3 resulting in cell death (type I cells) [9,10]. In type II cells (such as disc cells), there is a form of 'cross-talk' between the extrinsic and intrinsic systems (involving mitochondria) whereby CD95/Fas receptor activation and subsequent caspase-8 activity may not reach the threshold necessary to activate the common executioner caspases-3/7. Bid, the BH3 interacting domain death agonist, serves as a vital intermediary in the 'cross-talk' that can occur between the intrinsic and extrinsic pathways [8]. Bid activation results in degradation of the mitochondrial membrane by blocking the anti-apoptotic action of Bcl-2, an outer mitochondrial membrane protein [8,9]. This loss of Bcl-2-mediated mitochondrial homeostasis leads directly to mitochondrial swelling and rupture of the organelle and release of cytochrome c into the cytosol [8-10]. Within the cytosol cytochrome c is directly involved with the formation of the apoptosome and the activation of caspase-9 that in turn activates downstream effector caspase-3, -7 and -6 resulting in cell death [8]. In this study, we assessed the apoptotic pathway(s) involved in the IL-1β and FasL induced apoptosis of bovine NP cells under hypoxic conditions. The NP cellular and extracellular matrix (ECM) is a tightly regulated environment where homeostasis is maintained by the matrix metalloproteinases (MMPs), the ADAMTS family of enzymes (A disintegrin and metalloproteinase with thrombospondin motifs), tissue inhibitors of metalloproteinases (TIMPS), and regulation of inorganic pyrophosphate (PPi) transport by ANK [11,12]. ANK (progressive ankylosis/human homologue of progressive ankylosis) is a transmembrane protein encoded by the *ANK *gene that controls pyrophosphate levels in cells and tissues of joints. The MMP and ADAMTS enzymes function in a similar fashion to those in skin and cartilage, and in an integrated fashion, balance anabolic and catabolic processes within the framework of ongoing tissue remodeling [11,13]. Dysregulation of the activity of the MMPs, ADAMTS and TIMP enzymes results in increased catabolic activity and progression of DDD largely due to the influence of inflammatory cytokines, in particular IL-1β and the cell surface 'death receptor' Fas/CD95 [14,15]. The inflammatory cytokine IL-1β is widely regarded as a key factor in the progressive degeneration of the IVD NP and FasL has been shown to sensitize disc cells to Il-1β mediated apoptosis-resulting in a synergistic action upon disc cells that increases with further degeneration [15]. It has recently been demonstrated that with increasing disc degeneration the NP displays elevated ANK protein expression and these changes may be associated with hydroxyapatite deposition contributing to progression of degenerative disease [12]. ANK is also known to preserve the differentiated chondrocyte phenotype (a critical element in the development of both DDD and osteoarthritis). In the presence of IL-1ß, chondrocytes express less hypertrophic chondrocyte-specific markers and are thought to lose their differentiated chondrocyte phenotype [16].

The progressive loss of viable cells and ECM integrity within the IVD NP is a pivotal process that depends upon a number of factors including cellular metabolism, cell number, nutrition of the disc (endplate integrity and diffusion characteristics), disc cell phenotype, and age [17-19]. With respect to IVD cellular content, the persistence of notochordal cells within the disc NP of certain animals such as the NCD canine is thought to contribute uniquely to resistance to DDD. Here for the first time we demonstrate that soluble factors secreted by notochordal cells (notochordal cell conditioned medium or 'NCCM') are capable of protecting NP cells from matrix protein degradation and pro-inflammatory cytokine secretion induced by IL-1ß and FasL. Furthermore, IL-1ß and FasL-mediated cell death is inhibited by NCCM via the inhibition of activated caspases -9 and -3. The ability to harness the restorative properties of the notochordal cell may lead to novel, molecular therapies in the treatment of DDD.

## Materials and methods

### Bovine NP cells

We obtained NP cells from the tails of 3-year old steers (bovine caudal discs) to be used as the 'target' cells in our experiments. Typically we harvested the NP from five to six bovine tails and enzymatically digested the NP overnight in order to obtain the cells. We removed the nucleus pulposus from the caudal discs of five to six bovine tails and after overnight digestion expanded the cells through two passages and then after suspension in 10% DMSO with 90% FBS (4 × 10^6 ^cells/mL) froze the cells at -80°C. We also developed bovine NP cell conditioned medium (BCCM) in exactly the same fashion as we obtained bovine NP 'target' cells but in the case of BCCM cultured the bovine NP cells within alginate beads.

### Non-chondrodystrophic canine notochordal cells

We obtained the NP from the lower thoracic and entire lumbar IVDs from 11 non-chondrodystrophic canines (6 in the first series of serum-free experiments and 5 in the second 2% NCCM experiments) and after overnight digestion seeded the cells within alginate beads for subsequent culture.

### Conditioned medium

We generated conditioned medium from both non-chondrodystrophic canine notochordal cells (NCCM) and bovine NP cells (BCCM). We then tested the ability of bovine NP conditioned medium or canine notochordal cell conditioned medium to protect bovine NP cells from cell death in the presence of IL-1β+Fas ligand (FasL). We also determined the ability of NCCM to protect the expression of salient extracellular matrix genes and other molecules under these same *in vitro *conditions using qRT-PCR methods. We used flow cytometry and activated caspase assays to determine protection from cell death and to discern the apoptotic pathways involved.

### Tissue harvest

#### Non-chondrodystrophic canine discs

A total of 11 dogs was used in the experiments; 6 animals in the first series of serum-free experiments and 5 in the second 2% NCCM experiments. All animals were obtained in collaboration with a licensed animal facility and all practices were in accordance with the animal care policies and ethics approval board of Toronto Western Hospital. All animals were 8 to 12 months of age and had failed at adoption or were to be euthanized following biopharmaceutical intervention. Deep sedation was achieved using a combination of Acepromazine (Atravet-Aerst pharmaceuticals St. Laurent, Quebec, Canada 10 mg/mL) mixed with Xylazine 100 mg/mL (Xylomax-Bimeda-NHC Animal Health, Broomhill Road, Tallaght, Dublin 24, Ireland) at a combined dose of 1 mL/15 Kg body weight. Once deep sedation had occurred, euthanasia was accomplished using intravenous sodium pentobarbital (CDMV) (St. Hyacinthe, Quebec, Canada at a dose of 30 mL/kg body weight.

### Tissue culture conditions

#### Notochordal cell recovery

For each NCCM-related experiment, within one hour of euthanasia, the lumbar spine was removed from six dogs in one experiment and five dogs in another. After soft tissue dissection the spines were treated with Clidox™ and Betadine™, the NPs were removed aseptically from six to eight IVDs of each animal and transferred to Advanced Dulbecco's Modified Eagle Medium/F-12 (ADMEM/F-12) supplemented with 100 units penicillin/streptomycin (PS) (basal medium) (Life Technologies, Carlsbad, Cal, USA). After removal of any residual annulus fibrosus, the NP tissues were enzymatically digested sequentially over night according to established methods previously reported [4,5,20]. The following day, the cells were filtered with a 70 μm cell strainer (Falcon). This cell preparation was found to be close to 100% notochordal in content, recognized by the classic physaliferous, large vacuolated appearance of the cells.

##### Serum-free notochordal cell conditioned medium (NCCM)

In order to determine the effect of notochordal cell-secreted factors without any exogenous stimulatory factors, we developed serum-free NCCM. Briefly, canine NP cells were mixed with 1.2% sodium alginate at a density of 1.5 × 10^6 ^cells/mL. Using a 21G needle and 5 mL syringe, the cell/alginate mixture was drop-wise added to 4 mL of 102 mM CaCl_2 _at a density of 80 beads/well within 6-well plates (6 × 10^6 ^cells in total). The beads were allowed to polymerize for 15 minutes within the CaCl_2 _mixture and then each well containing the beads was washed 3 times with 150 mM phosphate buffered saline (PBS). Finally, the wells containing the alginate-notochordal cell beads were placed under hypoxic tissue culture (3.5% O_2_, 37°C and 5% CO_2_) with ADMEM/F12 supplemented with 8% fetal bovine serum (FBS) and 100 Units (U) penicillin/streptomycin (PS) for two days (complete medium). After two days of culture in complete medium, the alginate beads containing the notochordal cells were thoroughly washed with PBS (15 minutes per wash for two hours). The medium was replaced with 6 mL/well (3 wells total) of basal medium (FBS-deficient ADMEM/F-12 containing 100 U PS) and after three more days of hypoxic culture serum-free NCCM was harvested and used for subsequent tissue culture.

##### 2% FBS supplemented NCCM (2% NCCM)

In order to ensure that our caspase assay results would not be skewed by the notochordal cells depleting the available nutrients over the culture period, we generated 2% NCCM using basal medium supplemented with 2% FBS (2% basal medium) in order to provide a modest amount of nutrients to sustain the notochordal cells. For these experiments we obtained notochordal cells from five separate NCD canines, exactly as above and developed alginate beads containing notochordal cells at a density of 0.75 × 10^6 ^cells/mL in a volume of 3 mL/well (five wells). There is variable recovery of notochordal cells from NCD canines and from this series of animals there were fewer cells recovered than in the serum-free experiments. Therefore we reduced the concentration of the cells within the beads as well as the volume of medium used for these experiments in order to attempt to approximate the concentration of NCCM developed. The beads containing notochordal cells were cultured for two days in complete medium and then washed extensively using basal medium (4 washes × 15 minutes) and the medium replaced using 2% basal medium and cultured within 2% basal medium for three days before harvesting. We refer to these supernatants as 2% NCCM.

#### Bovine NP cells

Bovine NP cells (obtained earlier from caudal discs of five to six three-year old steers were released by enzymatic digestion, filtered, counted, plated and cultured under hypoxic conditions in T-75 flasks (CoStar-Corning Life Technologies, Corning, New York USA) in complete medium. The cells were allowed to expand to 70% confluence, harvested using Trypsin (Sigma-Aldrich Canada Ltd., Oakville, Ontario, Canada), and re-suspended in 90% FBS and 10% DMSO. Next the cells were sequentially frozen first at -80°C for two days and then transferred to liquid nitrogen. The day that the canine notochordal cells were harvested, we thawed the bovine NP cells in a 37°C water bath and after washing with complete medium the cells were plated and allowed to expand to 70% confluence at 37°C under hypoxic conditions of 3.5% O_2 _and 5% CO_2_. Coincident with the development of NCCM, the bovine NP cells were trypsinized, extensively washed with basal medium and re-seeded within 6-well plates at 0.5 × 10^6 ^cells/well and placed under hypoxia and allowed to expand for 3 days to 80% confluence using complete medium. Twenty-four hours prior to the NCCM-related experiments the plated bovine NP cells were extensively washed with basal medium and then cultured in basal medium in preparation for the NCCM experiments. For the 2% NCCM experiments, we initially thawed bovine NP cells and expanded them using complete medium, then washed them three times with 2% FBS supplemented basal medium in preparation for these experiments.

#### 2% FBS supplemented bovine NP conditioned medium (2%BCCM)

In order to be sure that any beneficial effects conferred by NCCM were not simply a cell-induced artifact, we compared the effects of 2% NCCM to conditioned medium made from bovine NP cells also supplemented with 2% FBS. Therefore we obtained bovine NP cells from the caudal discs of five to six three-year old steers, seeded them within alginate beads, cultured them within basal medium supplemented with 2% FBS to develop '2% BCCM'. We then used 2% BCCM in exactly the same fashion as 2% NCCM to test its effect upon bovine NP cells.

#### Induction of apoptosis

We induced apoptosis of monolayer cultured bovine NP cells by the addition of 10 ng/mL recombinant bovine IL-1β (Thermo Scientific RP-87269 Nepean, Ontario, Canada) plus 10 ng/mL recombinant human Fas-ligand (FasL) (Alexis Biochemical Alx-522-001 San Diego, CA USA)). We then evaluated cell death using flow cytometry (after 48 hours) and activated caspase assays (after 24 hours). These time points were chosen after optimization experiments.

#### Protection from apoptosis and degradation

In order to determine if factors secreted by notochordal cells could specifically inhibit apoptosis and degradation of bovine NP cells, we cultured bovine NP cells with NCCM, 2% NCCM, and 2% BCCM, all in the presence of IL-1ß+FasL. For controls, we cultured bovine NP cells with basal medium for the flow cytometry experiments or with basal medium supplemented with 2% FBS; in all cases identical doses of IL-1β, or IL-1β + FasL were used. All experiments were performed at 37°C under hypoxic conditions of 3.5% O_2 _and 5% CO_2_.

### Experimental assays

#### Flow cytometry

Three 6-well plates containing bovine NP cells that had achieved approximately 80% confluence were divided such that each well of a 6-well plate received 4 mL/well of basal medium only (0% FBS medium), 1 plate received basal medium plus 10 ng/mL IL-1β + FasL/well, and one plate received 4 mL of serum-free NCCM + IL-1β+FasL/well. All samples were cultured under hypoxic conditions for 48 hours following which we determined differential cell death using flow cytometry.

#### Caspase-3 validation

In parallel to the flow cytometry experiments, bovine NP cells were cultured in two additional 6-well plates. Six wells of one plate received the same volume of serum-free basal medium (4 mL) and six wells in the other plate received serum-free basal medium + IL-1β + FasL. These plates were then processed to evaluate the induction of apoptosis using the Apo-Alert activated caspase-3 assay.

#### Caspase -8, -9 and -3/-7 assays

Separate experiments were performed using basal medium supplemented with 2% FBS, 2% NCCM and 2% BCCM and the expression of activated caspases was determined using the Caspase-Glo assay (Promega, Madison Wis, USA). We chose the Caspase-Glo assay for these experiments because this assay requires far fewer cells/assay. The 2% NCCM was generated from notochordal cells obtained from six to eight discs from five canine spines and the 'target' bovine NP cells were obtained from the discs of five to six three-year old steers. In this way we repeated our cell-death assays using cells obtained from entirely separate pools of cells from groups of animals in this case using activated caspase assays.

#### Gene Expression

We obtained total RNA from the same sets of cultures from which we assessed cell death using flow cytometry (serum-free basal medium only (0% FBS medium), serum-free basal medium plus 10 ng/mL IL-1β+FasL/well, and serum-free NCCM+IL-1β+FasL/well. Briefly, after the 48-hour culture period total RNA was extracted from each of three wells for each condition using Trizol™ Life Technologies, Carlsbad, CA, USA) according to the manufacturer's recommendations. The RNA was purified, quantified using Nanodrop™ (Nepean, Ontario, Canada) and after reverse transcription, real-time RT-PCR evaluation for the genes of interest was performed.

### Evaluation of Cell Death

#### Flow Cytometry

We used flow cytometry (Becton Dickinson Dual Laser FacsCalibre (Mississauga, Ontario, Canada)) to evaluate the total extent of bovine NP cell death and apoptosis by incubating the cells in appropriate buffers and labeling with Propidium Iodide (PI) and Annexin-V (AV), (Sigma-Aldrich Canada Ltd., Oakville, Ontario, Canada) respectively, according to the manufacturer's instructions. AV is a protein that preferentially binds to phosphatidyl serine (PS) which undergoes specific changes during apoptosis and when conjugated to fluorescein isothiocyanate (FITC) it is capable of detecting early apoptosis via fluourescence imaging. PI is capable of entering the cell only when the plasma membrane has been dirsupted and is a non-specific marker of cell death which is often used together with AV to discriminate apoptosis from necrotic cell death. AV (to detect apoptotic cells) and PP (to detect dead/necrotic cells) were added to each sample according to the manufacturer's instructions and incubated at room temperature in the dark for 10 minutes. The cell harvest was filtered using 70 μm cell strainers (Falcon) just prior to flow cytometry analysis for which we used 'FacsCalibre'. Data analysis was performed using CellQuest Pro.

#### Flow Cytometry Optimization

**Figure 1:**
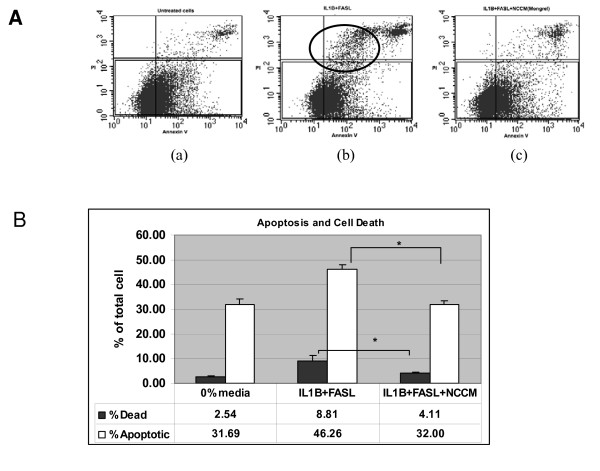
**Flow Cytometry Analysis of NCCM-Induced Protection from Apoptosis in NP Cells**. (A) Representative sample of flow cytometry analysis of bovine NP cell death (Propridium Iodide (PI) (Y axis) and Annexin V (X axis) staining. All experiments were performed in triplicate. NCCM was generated from notochordal cells from six canine specimens. **(a) **0% (basal medium) control, **(b) **IL-1β + FasL, **(c) **IL-1β + FasL + NCCM (b) The use of IL-1β + FasL causes an increase in the PI+ and Annexin V+ cells (upper right quadrant = dead cells) as compared to either (a) or (c). PI+ and Annexin V- = necrosis; PI + and Annexin V+ = apoptotic cell death. Treatment with NCCM (c) results in far fewer cells that are PI+ and Annexin V+ with an appearance that is almost equivalent to baseline (a). The black oval in (b) represents the cell population within the IL-1β + FasL treated cells that are 'rescued' by NCCM since these increasingly Annexin V+ and progressively PI+ cells are not apparent in the NCCM-treated groups. Triplicate experiments are presented in graphically in Figure 1 (b). **(B) **Summary of flow cytometry NP cell death assays (triplicate experiments as depicted in Figure 1 (a) above. The dark bars depict the percentage of all dead cells (Annexin V+ plus PI+) and the white bars depict the percentage of the total dead cells for each condition that died via apoptosis (Annexin V+ but PI-). There is little death in the untreated (0% (basal) media) cells but there is a marked increase in cell death when treated with IL-1β + FasL. This increase in both total cell death (from 2.5% to 8.8%) and apoptotic cell death (from 31.69% to 46.2%) is brought almost to baseline by the use of NCCM (apoptotic cell death is brought back to a level that is identical to the untreated cells). The differences between IL-1β + FasL and IL-1β + FasL + NCCM in terms of both total and apoptotic cell death are statistically significant at *P *= .016 and *P *= .0002 respectively (see asterisk indicating significance). Treatment with IL-1β + FasL + NCCM results in the most cell death as a function of apoptotic, not necrotic cell death. Abbreviations: IL-1β (Interleukin-1beta) FasL (Fas Ligand), NCCM (notochordal cell conditioned medium),

We adjusted the initial scatter plot to avoid small cells or sub-celluar debris and then subsequent gating around dead cells (AV+ and PI+ cells) determined from analysis of cells treated with distilled H_2_0. Our experimental data were obtained using the gating protocols we developed from the positive (distilled H_2_0) and negative (untreated cells) controls. Necrotic cells were interpreted as PI-positive and AV-negative and cells undergoing apoptosis as AV+ and PI-. Cells that were both PI+ and AV+ were considered to be mixed populations (apoptotic or necrotic). We used serum starvation and etoposide, known inducers of apoptosis as positive controls and verified using flow cytometry (AV+ and PI- cells) that serum starvation and 300 μM etoposide induces discriminate populations of AV+ and PI- populations. We pooled bovine NP cells obtained from the caudal discs of six animals and used these cells as the 'target cells'. NCCM was generated from pooled notochordal cells obtained from six to eight IVDs from each of six non-chondrodystrophic canines as detailed above. All treatments were performed in triplicate with cells incubated for 48 hours under hypoxia. The cells from each well were then collected individually by trypsinization (both suspended/dead cells plus monolayer cells were all collected for total cell recovery) and subjected to trypsin inactivation by two washes with complete medium, washed in ice cold PBS and re-suspended in 1 mL binding buffer (AnnexinV apoptosis detection kit, K101-100, Cedarlane (Burlington, Ontario, Canada)). Untreated cells re-suspended in DH_2_0 for 15 minutes prior to adding binding buffer were used as a positive control for dead cells. We quantified our results by determining the mean % AV+, PI- (apoptotic) cell and the mean %AV-, PI+ (necrotic) cell populations. For each experimental condition we calculated the percent of apoptotic cells as a function of the total percent of dead cells and expressed the results graphically (Figure 1). We determined the statistical significance for protection from apoptosis by the use of the T-test for the cells treated with IL-1β+FasL compared to the NCCM+ IL-1β+FasL conditions.

In order to ensure that our flow cytometry results were reliable in the detection of apoptotic events we evaluated the expression of caspase-3 in cells treated with complete medium only, as well as complete medium plus IL-1ß + Fas. These cells were then processed according to the manufacturer's protocol and assayed by flurometry (ApoAlert Caspase-3 Fluorescent assay kit, ClonTech Labs inc., #630215 (Mountainview California, USA). The caspase-3 assay detects an alteration in the emission spectra of fluorescence of 7-amino-4-trifluoromethyl coumarin (AFC). The emission spectra shift from 400 nm to 505 nm after the AFC-labeled caspase-3-specific substrate is proteolytically cleaved by the enzyme and releases free AFC. Thus fluorescence intensity at 505 nm is directly proportional to activated caspase-3 and is representative of apoptotic events. These optimization experiments validated that our flow cytometry assay can reliably detect apoptosis in NP cells (Additional file 1).

#### Activated caspase -8, -9 and -3/7 assays

Flow cytometry can discriminate between non-apoptotic and apoptotic cell death but does not define the mechanisms of such cell death or protection from apoptosis in the case of NCCM treated cells. Therefore, we examined the expression of activated caspases-8, -9 and -3/7 using the Caspase-Glo assay (Promega). This assay was selected because it required far fewer cells compared to the ApoAlert assay used to validate our flow cytometry data allowing the relatively small amounts of NCCM generated to be used in quadruplicate. Briefly, in this assay the caspase enzyme specific luminogenic-substrate is cleaved by active caspases in the cell lysate releasing a substrate for luciferase. We plated bovine NP cells in 96 well plates at a density of 1 × 10^4 ^cells/well. The bovine NP cells were then incubated overnight with complete media under our hypoxic conditions. The following day the cells were washed and incubated with basal medium for three hours prior to treatment. There were three treatment groups: (1) bovine NP cells cultured with 2% ADMEM/F12 and treated with IL-1ß+FasL, (2) bovine NP cells cultured with 2% BCCM in the presence of IL-1ß+FasL and (3) NP cells cultured with 2% NCCM in the presence of IL-1ß+FasL. All treatment groups have a final volume of 100 ul/well. After the 24-hour treatment period, 100 ul caspase reagent was added to each well according to the experimental design. For caspase -8 and -9, the proteosome inhibitor MH-132, 60 uM, was added to the caspase reagent just prior to use. The plates were then incubated at room temperature for 75 minutes and luminescence detected with Perkin Elmer Victor3 Multilabel coulter (Model 1420-050). For background controls, we measured control luminescence from wells containing treatment media and the caspase reagent but no cells (Av lum - Av background lum = Relative Luminescence). All assays were performed in triplicate and the results examined statistically using the T-test.

### Gene Expression Analysis

We wished to determine if serum-free NCCM could protect the expression of important extracellular matrix (ECM) genes as well as the pain- and inflammatory-related cytokine IL-6. We used real-time RT-PCR (qRT-PCR) to examine the expression of ECM genes, aggrecan, collagen type 2 and the cell-surface glycoprotein hyaluronic acid assembly site; CD44 and link protein. We also examined the expression of genes encoding matrix protecting factors such as TIMP-1, the principal matrix degrading enzymes MMP-3 and ADAMTS-4 and the expression of *IL-6 *by NP cells. Finally, we also examined the capacity of NCCM to affect the expression of the *Ank *gene by bovine NP cells treated with IL-1ß+FasL. We evaluated the expression of these genes in bovine NP cells cultured with basal medium, basal medium supplemented with IL-1ß, basal medium supplemented with IL-1ß+FasL and finally serum-free NCCM-treated NP cells also in the presence of IL-1ß+FasL.

#### qRT-PCR Optimization

Each primer pair was optimized for both optimal primer concentration and amount of cDNA in order to determine that amplification was within 15 to 25 amplification cycles. By this approach, we avoided non-linear/endpoint amplification data. We evaluated the expression profile for each primer pair used to ensure that there were no aberrant peaks present during the amplification phase of the PCR reaction. In addition to examining the expression of the various genes of interest when cultured with serum-free NCCM we also repeated our experiments using total RNA obtained from the 2% NCCM experiments.

Primers were obtained using previously published studies and established databases [21]. All other primers were designed using Primer Express 3.0 according to standard guidelines, based on bovine coding sequences of each gene of interest (Table 1). Each primer set was optimized to determine the optimal primer concentration, annealing temperature and cDNA concentration. Total RNA was extracted from cells using Trizol reagent (Sigma) according to the manufacturer's recommendations. For cDNA synthesis, reverse transcription was performed on 1 ug of total RNA sample using SuperScript II based reverse transcription system (Life Technologies, Carlsbad, California, USA) Invitrogen. The RT-PCR analysis was performed using the iCycler iQ5 RT-PCR detection system (BioRad Laboratories Mississauga, Ontario, Canada) and iQ SYBR green Supermix (BioRad Laboratories). Primer sets with equitable efficiencies were determined based on cDNA standard curves and were used for quantitative RT-PCR analysis using the delta-delta-Ct method [22]. We normalized the expression of the target genes using the constitutively expressed gene hypoxanthine guanine phosphoribosyltranferase (HPRT). The threshold for determining significant changes in the normalized expression of genes of interest was established at a 1.5-fold difference in comparison with control levels and replicates were statistically evaluated using the T-test with significance denoted by asterisks at *P *< .05 (unless otherwise noted).

**Table 1:** Real-time RT-PCR Primers, annealing temperatures and Accession Numbers of genes evaluated

Gene	Primer Sequence	Annealing Temp °C	Accession Number
Aggrecan F	CCTGAACGACAAGACCATCGA	58	U76615
Aggrecan R	TGGCAAAGAAGTTGTCAGGCT	58	
			
Collagen 2 F	AAGAAGGCTCTGCTCATCCAGG	58	X02420
Collagen 2 R	TAGTCTTGCCCCACTTACCGGT	58	
			
MMP3 F	CACTCAACCGAACGTGAAGCT	58	AF135232
MMP3 R	CGTACAGGAACTGAATGCCGT	58	
			
TIMP1 F	TCCCTGGAACAGCATGAGTTC	58	AF144763
TIMP1 R	TGTCGCTCTGCAGTTTGCA	58	
			
ADAMTS4 F	CCTGGCAACGAGGACTCAAC	58	NM_181667.1
ADAMTS4 R	GGGTAAACAGAATGGCTGTGTCA	58	
			
CD44 F	CGGGTTCATAGAAGGGCATGT	58	X62881.1
CD44 R	TTGTTCGCAGCACAGATGGA	58	
			
Link protein F	AAGCTGACCTACGACGAAGCG	58	NM_174288.1
Link protein R	CGCAACGGTCATATCCCAGA	58	
			
IL-6 F	CTGGGTTCAATCAGGCGAT	58	NM_173923.2
IL-6 R	CAGCAGGTCAGTGTTTGTGG		
			
ANKH F ANKH R	TCGGTGTGGACTTTGCCTTT ACTGGAACCGGGAAGAAGGA	58	NP_001103263
			

Fold changes in gene expression were determined by dividing the mean ΔΔCT value calculated for each gene of interest for the IL-1β+FasL culture experiments by the mean ΔΔCT obtained for IL-1β+FasL+NCCM for each gene of interest (DD-CT fold change = (IL1B+FASL)/(IL1B+FASL+NCCM) (Table 2).

**Table 2:** Normalized fold changes (HPRT) in gene expression by NP cells cultured in NCCM and treated with IL-1β+FasL

Gene	Normalized Fold Change in Gene Expression
Collagen Type 2	1.57 fold increase
Aggrecan	65.5 fold increase
Link Protein	54.5 fold increase
CD44 Receptor	1.76 fold increase
*MMP3*	1.53 fold decrease
*ADAMTS4*	3.4 fold increase
*TIMP1*	4.8 fold increase
*IL-6*	1.9 fold decrease
*Ank*	1.5 fold decrease
	

'Fold change' was calculated by dividing the mean ΔΔCT value for each gene of interest treated with IL-1β + FasL/mean ΔΔCT for cells treated with IL-1β + FasL + NCCM. The gene expression data which is presented graphically in Figures 4 to 6 is normalized to basal medium conditions (bovine NP cells treated with basal medium without the addition of IL-1β or IL-1β + FasL. Abbreviations: CD44 (cluster of differentiation 44), MMP3 (matrix metalloproteinase-3), ADAMS4 (A disintegrin and metalloproteinase with thrombospondin motifs 4), TIMP-1 (tissue inhibitor of matrix metalloproteinase-1), IL-6 (interleukin-6), Ank (Ank gene).

## Results

### IL-1β and FasL mediated apoptosis in hypoxic bovine NP cell culture can be rescued by NCCM

We induced apoptosis of monolayer cultured bovine NP cells by the addition of 10 ng/mL recombinant bovine IL-1β plus 10 ng/mL recombinant human Fas-ligand (FasL) and evaluated cell death by flow cytometry (after 48 hours) and by the caspase assays (after 24 hours). These time points were chosen after optimization experiments. AV and PI labelled cells were quantified using flow cytometry allowing discrimination between viable/intact cells (AV-PI-), early apoptotic (AV+PI-) and late apoptotic or necrotic cells (AV+PI+). Cells were harvested from each of three conditions and after analysis using flow cytometry the percentage of dead cells in each condition was determined. Next from the dead cell populations the percentage of dead cells that were considered apoptotic was expressed as a function of the total percentage of dead cells (Figure 1). Treatment with NCCM resulted in a significant reduction in PI/AV positive cells that was similar to baseline levels with the rate of apoptotic cells indistinguishable from baseline (Figure 1, Panel c). We used an activated caspase-3 fluorometric assay to evaluate parallel bovine NP cells treated with IL-1β+FasL and found that this treatment induced apoptosis as demonstrated by the detection of activated caspase-3 (Additional File 1 Figure S1).

**Figure 2:**
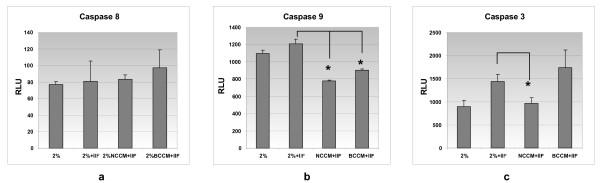
**Activated caspase assays for NP cell apoptosis**. **(a) **Activated caspase-8 activity for all experimental conditions; there is marginally increased activity in NCCM + IF and further elevated activity for BCCM but no suppression of activated caspase-8 activity for any of the treatment groups. **(b) **Activated caspase-9 activity is upregulated in the 2% +IF group and markedly down-regulated by NCCM (*P *< .01) and also (although to a lesser extent) by BCCM (*P *< .01). **(c) **Levels of activated caspase-3 for 2%, 2% + IL-1β + FasL, NCCM + IL-1β + FasL and BCCM + IL-1β + FasL treated cells. Here the use of NCCM in the presence of IL-1β + FasL reduces activated caspase-3 activity to almost baseline - precisely as demonstrated using flow cytometry and AnnexinV and PI labeling. Interestingly, there is no protection from apoptosis using conditioned medium obtained from bovine NP cells (BCCM) indicating that anti-apoptotic signaling is uniquely conferred by NCCM (*P *< .01). The suppression of both activated caspase-3 and -9 by treatment with NCCM indicates an anti-apoptotic effect of NCCM upon NP cells in the presence of IL-1β + FasL that is not accounted for by the other treatment groups. RLU (depicted on the 'Y' axis) is the fluorometric activity that occurs in the presence of caspase activation that releases a substrate for luciferase that in turn is determined using fluorometric methods. In each case 2% NCCM was generated from pooled notochordal cells obtained from the IVDs of five non-chondrodystrophic canines (eight to nine discs/animal). Bovine NP cells were obtained from the caudal discs of five to six three-year old steers. The 2% BCCM was generated from a separate pool of bovine caudal discs similarly obtained, seeded within alginate beads and conditioned medium developed over three days. Abbreviations: IL-1β (Interleukin-1beta) FasL (Fas ligand), NCCM (notochordal cell conditioned medium), BCCM (Bovine Cell Conditioned Medium), NP cells (nucleus pulposus cells), RLU (Relative Luminescence Units).

Flow cytometry can discriminate between non-apoptotic and apoptotic cell death but does not define the mechanisms of such cell death or protection from apoptosis in the case of NCCM treated cells. In order to determine the mechanisms underlying NCCM-mediated anti-apoptotic effects, we measured activated caspase activity of bovine NP cells treated with 2% NCCM and 2% BCCM versus basal medium supplemented with 2% FBS in the presence of IL1-β+FasL. There are no differences in the expression of activated caspase-8 in any of the treatment groups (Figure 2(a)). Both 2% NCCM and 2% BCCM significantly suppressed the expression of activated caspase-9 activity (both *P *< 0.01; Figure 2(b)). However, only 2% NCCM (but not 2% BCCM) significantly suppressed the expression of caspase-3/7 in the IL1β and FasL treated bovine NP cells (*P *< 0.01; Figure 2(c)). As activated caspase-3/7 are downstream effectors leading to apoptosis, only 2% NCCM has the factors to partially rescue IL1-β and FasL mediated apoptosis in bovine NP hypoxic cultures.

### Impact of NCCM on genes encoding structural proteins and modulators of the extracellular matrix

#### Structural protein gene regulation

**Figure 3:**
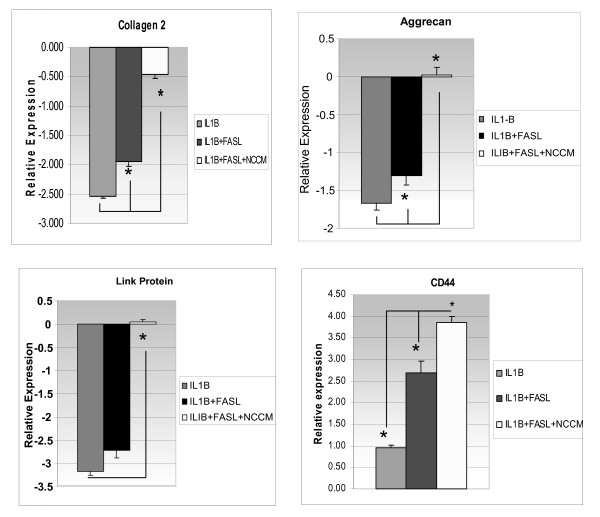
**Quantitative Real-time PCR (qRT-PCR) relative gene expression for collagen type 2, Aggrecan, Link Protein and the CD44 receptor expressed by bovine NP cells**. The expression of each gene represented is expressed relative to basal (untreated) conditions and is normalized to the constitutively expressed gene *HPRT*. Reading each graph from left to right is the relative fold gene expression of NP cells treated with IL-1β (grey bar), IL-1β + FasL (black bar), cells treated with IL-β + FasL + NCCM (white bar). NCCM was developed from the NPs from six dogs, the NP cells from the NPs of five to six bovine caudal discs cells pooled together. All qRT-PCR experiments were repeated in quadruplicate and are presented with standard error calculations performed for all conditions (statistical significance is *P *< .05). Abbreviations: qRT-PCR (quantitative Real-Time Polymerase Chain Reaction), CD44 (cell surface glycoprotein cluster of differentiation 44), HPRT (Hypoxanthine-guanine phosphoribosyltransferase), NP cells (nucleus pulposus cells), IL-1β (Interleukin 1 beta), FasL (Fas Ligand), NCCM (notochordal cell conditioned medium).

**Figure 4:**
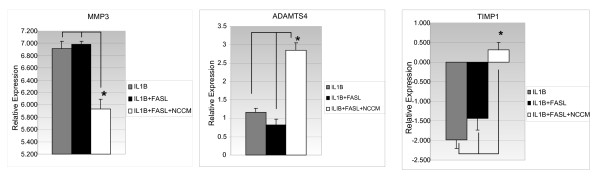
**Relative gene expression for MMP-3, ADAMTS-4, TIMP-1 (matrix remodeling genes) expressed by bovine NP cells (real-time PCR (qRT-PCR)**. Reading from left to right on each graph the grey bar reflects NP cells treated with IL-1β, the black bar is treatment with IL-1β + FasL, and the white bar NP cells treated with IL-1β + FasL + NCCM. As in Figure 3 each gene represented is expressed relative to basal (untreated) conditions and normalized to the constitutively expressed gene *HPRT*. NCCM was developed from notochordal cells obtained from the NPs of six non-chondrodystrophic canines and pooled together. Bovine NP (target cells) were collected from bovine caudal discs of five animals and pooled together. All qRT-PCR experiments were repeated in quadruplicate and are presented above with standard error calculations performed for all conditions. The statistical significance for NCCM-treated cells relative to IL-1β + FasL is indicated by the asterisk and indicator bars and is *P *< .05. Abbreviations: MMP-3 (matrix metalloproteinase-3), ADAMTS-4 (A disintegrin and metalloproteinase with thrombospondin motifs 4), TIMP-1 (tissue inhibitor of matrix metalloproteinase 1), IL-1β (Interleukin 1 beta), FasL (Fas ligand), NCCM (notochordal cell conditioned medium), qRT-PCR (quantitative real-time polymerase chain reaction).

The use of NCCM in cultures co-treated with IL-1β had a significant effect upon genes that encode structural proteins as well as the inhibitors and activators of extracellular matrix remodeling (Figures 3 and 4). We have presented our results relative to basal conditions (cells treated with basal medium without IL-1β or FasL). Consistent with past reports, aggrecan gene expression was decreased with the use of ILl-1β and IL-1β+FasL [14,15,23,24]. However, NCCM up-regulated aggrecan gene expression and demonstrated a markedly increased 65.5-fold difference compared to basal medium conditions (Table 2). Interestingly, NCCM also increased the expression of the gene encoding link protein to a 54.5-fold increase over baseline. NCCM up-regulated CD44 expression by 1.76-fold and inhibited the down-regulation of collagen 2 induced by IL-1β+FasL by 1.76-fold (Table 2).

#### Extracellular matrix remodeling and anti-inflammatory cytokines

The expression of extracellular matrix remodeling genes was protected by the use of NCCM. MMP3 gene expression is up-regulated by IL-1β and IL-1β+FasL but the gene expression of this catabolic enzyme is reduced by 1.5-fold by NCCM. The activity of the MMP inhibitor TIMP-1 is up-regulated by NCCM 4.8-fold and a 3.4-fold increase was observed in the expression of ADAMTS4 (Table 2) [25-27].

The use of IL-1β+FasL led to a strong increase in the expression of the IL-6 gene but the addition of NCCM to the cultures markedly down-regulated IL-6 gene expression by almost 50% (1.9-fold reduction).

#### *Ank *gene expression and NP cell phenotype

**Figure 5:**
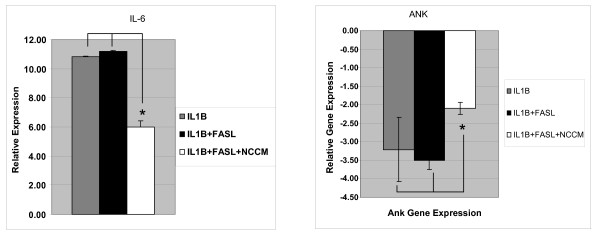
**Bovine NP cell expression of IL-6 and Ank genes (qRT-PCR)**. **(a) **Expression of IL-6 gene by NP cells treated with IL-1β, IL-1β + FasL, and IL-1β + FasL + NCCM. IL-1β, IL-1β + FasL induce a dramatic increase in the expression of the gene encoding IL-6; however, the use of NCCM results in a dramatic reduction of NP cell expression of IL-6. **(b) **NP *Ank *gene expression is significantly down-regulated by the use of IL-1β, IL-1β + FasL; however, this down-regulation is strongly attenuated by NCCM. NCCM was developed from the IVDs of six non-chondrodystrophic canines and pooled together. Bovine NP (target cells) were collected from bovine caudal discs of five animals and pooled together. All qRT-PCR experiments were repeated in quadruplicate and are presented above with standard error calculations performed for all conditions. The statistical significance for NCCM treated cells relative to IL-1β + FasL is indicated by the asterisk and indicator bars and is *P *< .05. Abbreviations: IL-6 (Interleukin-6), qRT-PCR (quantitative real-time polymerase chain reaction), NCCM (notochordal cell conditioned medium), IL-1β (Interleukin 1 beta), FasL (Fas ligand), NCCM (notochordal cell conditioned medium), qRT-PCR (quantitative real-time polymerase chain reaction).

The expression of ANKH (human homolog of *Ank*) is increased with increased amounts of human IVD degeneration suggesting that the ANK pathway may play a key role in the homeostatic regulation of the IVD [11,28]. Therefore we were interested in possible changes induced in NP cell expression of the *Ank *gene in our system. In keeping with prior studies on articular cartilage, down-regulation of the *Ank *gene expression was observed after the addition of IL-1β and IL-1β+FasL. This process was inhibited by NCCM by 1.5-fold (Figure 5) (Table 2).

## Discussion

The hallmark of progressive DDD is the inability of NP cells to maintain normal homeostatic tissue remodeling under the influence of increased cytokine and death receptor expression [11,12,18,26]. A number of studies have strongly implicated IL-1β+FasL as key molecules in the progression of DDD [11]. The purpose of this study was to determine the ability of NCCM to (1) counteract the deleterious gene expression changes induced by IL-1β+FasL *in vitro*; and (2) inhibit apoptosis of NP cells and determine the signaling pathways involved. Here we demonstrate for the first time that soluble factors secreted by notochordal cells strongly protect NP cells from IL-1β+FasL induced degeneration and apoptosis. Of significance NP cell apoptosis is inhibited by NCCM and is mediated in a caspase-dependent manner via suppression of activated Caspase -9 and -3/-7.

### Gene expression which leads to the protective effect of NCCM

Progressive DDD is associated with increased expression of IL-1β [14,15,23,29,30] and FasL. We showed that IL-1β- and FasL-induced bovine NP cells under hypoxic conditions provide an appropriate system for the dissection of mechanisms underlying IVD degeneration. Our results indicated that changes induced by IL-1β are further stimulated by the addition of FasL. Thus, we used this *in vitro *system to identify the deleterious genes which can be counteracted by NCCM. Our RT-PCR results showed that the protective effect of NCCM is mainly due to (1) the inhibition of the transcript expression of a proinflammatory cytokine, IL-6, and a major collagenase, MMP-3 (likely via upregulation of TIMP-1, a natural inhibitor of MMP-3); and (2) the homeostatic regulation of matrix synthesis involving the maintenance of aggrecan (likely via increased expression of *ADAMTS4*), link protein and CD44 expression.

#### 1. IL-6

It has been shown that IL-6 expression is increased in DDD and is correlated with painful degenerative discs [31,32]. As reported by others, treatment of NP cells with IL-1β alone or together with FasL led to upregulation of *IL6 *transcript expression [33]. The use of NCCM downregulated its expression by more than 50%, suggesting that factors in NCCM, in part, inhibit the inflammatory aspect of disc degeneration.

#### 2. MMP3 (stromelysin)

MMP-3 is a major collagenase involved in the irreversible degradation of collagen [24]. TIMPs bind to MMPs and protect proteoglycan degradation [31]. Our results showing up-regulation of *TIMP-1 *and down-regulation of *MMP-3 *transcripts, respectively, suggested that NCCM conferred an anti-degradative effective on the IL-1β- and FasL-treated MP cells.

#### 3. ECM homeostasis

As part of the degenerative process, degraded link protein and proteoglycans accumulated in NPs much faster than in articular cartilage [34,35]. Recently, aggrecan degradation and turnover has been shown to be a reversible event, indicating there might be a window of opportunity whereby proteoglycan degradation could be prevented before the irreversible MMP-mediated degradation occurs [21,22]. It is possible to restore disc height and increase T2 MRI signals after trophic factors delivery to the disc following initial chemonucleolysis and/or needle puncture [35,36].

We and others showed that IL-1β induced NP cells to up-regulate the transcript expression of molecules involved in ECM modeling (*ADAMTS-4 *and *CD44*) [14,23]. Our results showing that NCCM upregulated *ADAMTS-4 *and *CD44 *expression may support the notion that some NCCM factors stimulate the remodeling process to preserve collagen type 2, aggrecan and link protein. The ADAMTS family of aggrecanases (notably ADAMTS-4 in the disc NP) functions in a reversible fashion to degrade the aggrecan core protein [24]. Aggrecan plays an important role in the hydration of NP, mainly by binding to hyaluronic acid and being stabilized by link protein [32,33]. Our observation that NCCM maintained the expression of aggrecan, CD44 and link protein reiterated that part of the protective effects of NCCM is mediated via maintenance of ECM homeostasis.

#### 4. *Ank*/ANKH

There is emerging evidence that Ank/ANKH plays a role in IVD degeneration. The best documented ANK function involves regulation of inorganic pyrophosphate (PPi) transport across the cell membrane [16]. It is also involved in differentiation of cell lineages such as osteoblast and osteoclasts [36]. In rat NP cells, hypoxia regulates *Ank *gene expression [12] and *ANKH *expression is increased in degenerated human IVDs [12], leading to the hypothesis that loss of homeostatic regulation of PPi is involved in degeneration and calcification in the IVD. In another study, IL-1β-treated chondrocytes *in vitro *resulted in a loss of differentiated chondrocyte phenotype (diminished *Sox2, aggrecan *and *collagen II *transcripts) and diminished *Ank *expression [16]. We noted a similar effect upon bovine NP cells treated with IL-1β with or without FasL in which reduced *Ank *gene expression occurred and this down-regulated *Ank *expression was attenuated by NCCM. However, our experiments were short term only (24 to 48 hours) and were performed on single cell *in vitro *cultures devoid of the complexities and feedback from the extracellular matrix and long-term degenerative changes that occur *in vivo*. It remains to be investigated whether NCCM contains sufficient PPi levels that contribute partly to its protective effect. Nonetheless, our *in vitro *results showing down regulation of *Ank *expression in treated NP cells (when taken together with down regulated aggrecan and collagen type 2 gene expression) appear to be consistent with a loss of phenotype maintenance known to occur in chondrocytes treated with IL-1β. The notably partial rescue of *Ank *gene expression by NCCM is suggestive of a homeostatic/protective effect conferred upon NP cells by the components of notochordal cell-secreted factors.

### Mechanistic dissection of the protective effect of NCCM on NP cell apoptosis induced by IL-1β and FasL

**Figure 6:**
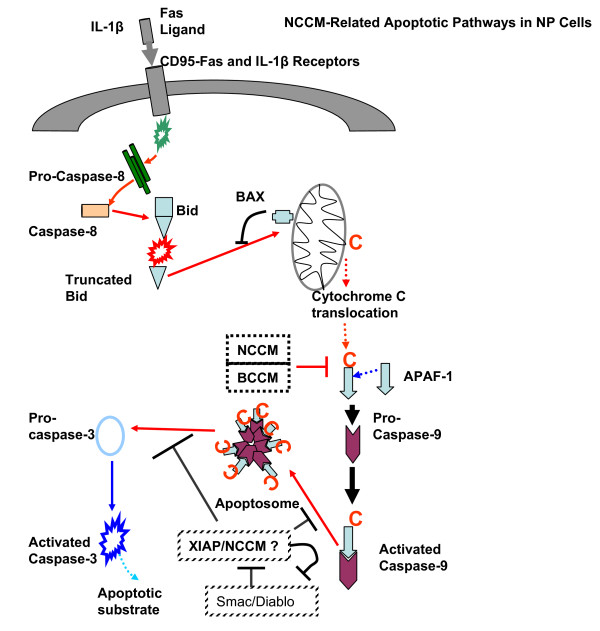
**Schematic of mitochondrial pattern of apoptosis in type II Nucleus pulposus cells**. 1. Caspase-9 activation via cross talk from Fas-receptor and caspase-8 induced Bid-T-Bid induction of cytochrome c release by mitochondria and downstream apoptosis ultimately via activation of caspase-3. 2. Potential areas where NCCM/BCCM (dotted boxes) may function upstream of activated caspase-9. 3. Potential mechanisms of action of NCCM downstream of activated caspase-9 via may function to inhibit activated caspase-9 and its function within the apoptosome, inhibit XIAP-like activity or by anti-Smac/Diablo activity resulting in inhibition of pro-apoptotic function of Smac/Diablo. 4. NCCM may function or contain XIAP or XIAP-like inhibitors of apoptosis or it may function to inhibit activated caspase-9 and its function within the apoptosome. Abbreviations: NCCM (notochordal cell conditioned medium), BCCM (bovine cell conditioned medium), XIAP (X-linked inhibitor of apoptosis protein), Smac (small mitochondria-derived activator of caspases), Diablo (direct IAP binding protein with low pI).

Figure 6 provides a synopsis of the signaling pathways involving the CD95/Fas death receptor (extrinsic system) and the intrinsic pathway mediated by the mitochondria. Type II cells (such as disc cells) utilize both the extrinsic and intrinsic pathways. We took advantage of the upstream/downstream effector caspases in the FasL mediated apoptotic pathways to evaluate the step(s) in which the NCCM factors exert their protective effects. Caspase-8 is upstream of the pathway close to the Fas/CD95 receptor; and caspase-9 and -3 are downstream of the mitochondrial effects while caspase-3 is the apoptosis executioner. The presence of NCCM in the IL-1β- and FasL-treated NP cells inhibited activation of both caspase-9 and -3 while BCCM inhibited only activation of caspase-9. This significant result positioned the protective effects of NCCM factors to events downstream of caspase-9 actions (Figure 6). Pro-caspase-9 and APAF-1 form the 'apoptosome', a process mediated by cytochrome c (C*, Figure 6) which is translocated from the mitochondria. Formation of the apoptosome leads to activation of caspase-3 and this step was inhibited by NCCM (but not BCCM) in our IL-1β- and FasL-treated NP cells. Our results are in keeping with the results of others [9] showing that Bid, cytochrome-c, and activated caspases-9 and -3 were robustly detected in all herniated NP tissues examined (*n *= 32 cases) while little caspase-8 activity was detected. We recognize that apoptotic signaling downstream of activated caspase-9 involves complex interactions between such mediators of Smac/Diablo and X-linked inhibitor of apoptosis proteins (XIAP) that control activated caspase-3 signaling [37]. Components within NCCM may function in similar ways to these naturally occurring inhibitors of apoptosis although at present these mechanisms are unknown. Studies to examine the role played by NCCM factors in reducing activated caspase-3 are ongoing.

### Study limitations

#### Gene Expression

'Fold changes' in gene expression are dependent upon threshold calculations (ΔCt and ΔΔCt), therefore very small denominators could over-estimate such fold changes and must be interpreted with some caution. However, we observed similar gene expression changes for these two genes in entirely separate experiments using cells pooled together from completely independent animals. Therefore, when taken together with our previous findings that NCCM up-regulates NP aggrecan expression, it does appear that there is a strong protection of aggrecan and link protein conferred by the components of NCCM.

#### NCCM 'dose'

We developed NCCM from two entirely separate pools of non-chondrodystrophic canine notochordal cell sources. In the first set of serum-free NCCM we used 1.5 × 10^6 ^cells/mL alginate and in the second we used half that concentration. We have determined through past experience that a dose-dependent relationship exists between the concentration of notochordal cells and the biological effect of NCCM [4] and have considerable experience generating NCCM. In the second set of 2% NCCM experiments where we developed NCCM from 0.75 × 10^6 ^cells/mL alginate we reduced the volume of medium used to generate the NCCM by 50% from 6 to 3 mL thereby increasing the 'concentration' of the NCCM in an attempt to mitigate against the lower notochordal cell harvest from those animals. We observed similar gene expression effects upon the bovine NP cells (data not shown) and observed suppressed activated caspase-3 activity verifying that just as with the serum-free NCCM, the 2% NCCM retained its anti-apoptotic activity.

## Conclusions

We have demonstrated in this study that NCCM protects against NP apoptosis via suppression of activated caspase-9 and -3/7. Possible mechanisms include stabilization of the mitochondrial membrane via inhibition of Bcl-2 activity, Bid activation or through the P53 growth factor-related pathway. It may be that components of NCCM suppress the formation of the apoptosome and in so doing suppress the formation of activated caspase-3 thereby preventing apoptotic cell death. The novel finding of the activation of the *Ank *gene by NCCM under degenerative/death-inducing conditions suggests a possible role for this gene in the maintenance of NP cell phenotype. These areas are under examination by our group.

The results of the present study provide evidence *in vitro *that notochordal cell-secreted soluble factors provide essential molecular signals that mediate apoptosis and degradation of NP cells induced by IL-1β+Fas-L. Harnessing the regenerative capacity of these cells and the important factors they secrete may lay the cornerstone of biological therapy for the treatment of degenerative disc disease.

## Abbreviations

ADAMTS: a disintegrin and metalloproteinase with thrombospondin motifs; ADMEM/F-12: Advanced Dulbecco's Modified Eagle Medium with F-12 supplement; AFC: 7-amino-4-trifluoromethyl coumarin; ANKH: a transmembrane protein encoded by the *ANKH *gene that controls pyrophosphate levels in cells and tissues of joints although it is found in other tissues. A mutation at the (ank) locus results in generalized, progressive arthritis and joint destruction/ankylosis; AV: annexin-V; BCCM: bovine NP cells; Bcl-2: B-cell lymphoma 2; Bid: BH3 interacting domain death agonist; CD: chondrodystrophic canine; CD95: cluster of differentiation 95 (also known as Fas-receptor); DDD: degenerative disc disease; DMEM: Dulbecco's modified Eagle's medium; ECM: extracellular matrix; FasL: soluble Fas receptor ligand; Fas Receptor: receptor for Fas-ligand also known as CD95; FBS: fetal bovine serum; IL-1ß: interleukin-1 beta; IL-6: interleukin-6; IVD: intervertebral disc; MMP: matrix metalloproteinase; NCCM: notochordal cell conditioned medium; NCD: non-chondrodystrophic canine; NP: nucleus pulposus; PBS: phosphate-buffered saline; PI: propridium iodide; PPi: inorganic pyrophosphate; PS: penicillin/streptomycin; RT-PCR: real time polymerase chain reaction; TIMPS: tissue inhibitors of metalloproteinases.

## Competing interests

The authors declare that they have no competing interests.

## Authors' contributions

WME formulated and designed experiments and was involved with all experimental data collection, performance of the experiments and was senior author on writing manuscript. DI was responsible for all aspects of performance of the experiments, optimization of the primers used, senior technical aspects, caspase assays and manuscript preparation. FT, RI and MF provided assistance with experimental design and manuscript preparation. All authors read and approved the final manuscript.

## Supplementary Material

Additional file 1 **Figure S1: Activated caspase-3 activity in NP cells treated with IL-1β + FasL for 24 and 48 hours (expressed relative to untreated cells)**. There is a clear and significant induction of cleaved/activated caspase-3 in the 48-hour treatment group verifying the induction of apoptosis. This is the same trend observed with fluorescent activated cell sorting analysis using Annexin V and Propidium Iodide. (Y axis refers to luminescence units/hour). Click here for file

## References

[B1] Goetzel RZ Hawkins K Ozminkowski RJ Wang S The health and productivity cost burden of the "top 10" physical and mental conditions affecting six large U.S. employers in 1999 J Occup Environ Med 2003 45 5 14 10.1097/00043764-200301000-00007 12553174

[B2] Aguiar DJ Johnson SL Oegema TR Notochordal cells interact with nucleus pulposus cells: regulation of proteoglycan synthesis Exp Cell Res 1999 246 129 137 10.1006/excr.1998.4287 9882522

[B3] Oegema TR Jr The role of disc cell heterogeneity in determining disc biochemistry: a speculation Biochem Soc Trans 2002 30 839 844 12440929 10.1042/bst0300839

[B4] Erwin WM Inman RD Notochord cells regulate intervertebral disc chondrocyte proteoglycan production and cell proliferation Spine 2006 31 1094 1099 10.1097/01.brs.0000216593.97157.dd 16648742

[B5] Erwin WM Ashman K O'Donnell P Inman RD Nucleus pulposus notochord cells secrete connective tissue growth factor and upregulate proteoglycan expression by intervertebral disc chondrocytes Arthritis Rheum 2006 54 3859 3867 10.1002/art.22258 17136753

[B6] Nesrin Ozoren El-Deiry Wafik S Defining characteristics of types I and II apoptotic cells in response to TRAIL Neoplasia 2002 4 551 557 10.1038/sj.neo.7900270 12407450 PMC1503670

[B7] Krammer Peter H CD95's deadly mission in the immune system Nature 2000 407 789 795 10.1038/35037728 11048730

[B8] Yin X-M Wang K Gross A Zhao Y Zinkel S Klocke B Roth KA Korsmeyer SJ Bid-deficient mice are resistant to Fas-induced hepatocellular apoptosis Nature 1999 400 886 891 10.1038/23730 10476969 PMC13623213

[B9] Jong-Beom Park Jin-Kyung Lee Sung-Jin Park Ki-Won Kim Riew KD Mitochondrial involvement in Fas-mediated apoptosis in human lumbar disc cells J Bone Joint Surg 2005 87-A 1338 1342 10.2106/JBJS.D.02527 15930545

[B10] Hengartner MO The Biochemistry of Apoptosis Nature 2000 407 770 776 10.1038/35037710 11048727

[B11] Le Maitre C Pockert A Buttle DA Freemont AJ Hoyland JA Matrix synthesis and degradation in human intervertebral disc degeneration Biochem Soc Trans 2007 35 652 655 10.1042/BST0350652 17635113

[B12] Skubutyte R Markova D Freeman TA Anderson GD Dion AS Williams CJ Shapiro IM Risbud MV Hypoxia-inducible factor regulation of ANK expression in nucleus pulposus cells: possible implications in controlling dystrophic mineralization in the intervertebral disc Arthritis Rheum 2010 62 2707 2715 10.1002/art.27558 20496369 PMC3065237

[B13] Hornebeck W Down-regulation of tissue inhibitor of matrix metalloproteinase-1 (TIMP-1) in aged skin contributes to matrix degradation and impaired cell growth and survival Pathol Biol (Paris) 2003 51 569 573 10.1016/j.patbio.2003.09.003 14622947

[B14] Le Maitre CL Freemont AJ Hoyland JA The role of interleukin-1 in the pathogenesis of human intervertebral disc degeneration Arthritis Res Ther 2005 7 R732 45 Epub 10.1186/ar1732 15987475 PMC1175026

[B15] Cui LY SL L Ding Y Huang DS Ma RF Huang WG Hu BS Pan QH IL-1 beta sensitizes rat intervertebral disc cells to Fas ligand mediated apoptosis in vitro Acta Pharmacol Sin 2007 28 1671 1676 10.1111/j.1745-7254.2007.00642.x 17883956

[B16] Cailotto F Sebillaud S Netter P Jouzeau JY Bianchi A The inorganic pyrophosphate transporter ANK preserves the differentiated phenotype of articular chondrocytes J Biol Chem 2010 285 10572 10582 10.1074/jbc.M109.050534 20133941 PMC2856265

[B17] Urban JP The role of the physicochemical environment in determining disc cell behaviour Biochem Soc Trans 2002 30 858 864 12440933 10.1042/bst0300858

[B18] Urban JP Smith S Fairbank JC Nutrition of the intervertebral disc Spine 2004 29 2700 2709 10.1097/01.brs.0000146499.97948.52 15564919

[B19] Shirazi-Adl A Taheri M Urban JP Analysis of cell viability in intervertebral disc: effect of endplate permeability on cell population J Biomech 2010 43 1330 1336 10.1016/j.jbiomech.2010.01.023 20167323

[B20] Erwin WM Las Heras F Islam D Fehlings MG Inman RD The regenerative capacity of the notochordal cell: tissue constructs generated in vitro under hypoxic conditions J Neurosurg Spine 2009 10 513 522 10.3171/2009.2.SPINE08578 19558283

[B21] Fitzgerald JB Jin M Grodzinsky AJ Shear and compression differentially regulate clusters of functionally-related temporal transcription patterns in cartilage tissue-supplemental material J Biol Chem 2006 281 24095 24103 10.1074/jbc.M510858200 16782710

[B22] Nomura S Yamaguchi MO Wang TC Lee JR Goldenring JR Alterations in gastric mucosal lineages induced by acute oxyntic atrophy in wild-type and gastrin-deficient mice Am J Physiol Gastrointest Liver Physiol 2005 288 362 375 10.1152/ajpgi.00160.2004 15647607

[B23] Le Maitre CL Hoyland JA Freemont AJ Catabolic cytokine expression in degenerate and herniated human intervertebral discs: IL-1beta and TNFalpha expression profile Arthritis Res Ther 2007 9 R77 10.1186/ar2275 17688691 PMC2206382

[B24] Pockert AJ Richardson SM Le Maitre CL Lyon M Deaking JA Buttle DA Freemont AJ Hoyland JA Modified expression of the ADAMTS enzymes and tissue inhibitor of metalloproteinases 3 during human intervertebral disc degeneration Arthritis Rheum 2009 60 482 491 10.1002/art.24291 19180493

[B25] Patwari P Gao G Lee JH Grodzinsky AJ Sandy JD Analysis of ADAMTS4 and MTW-MMP indicates that both are involved in aggrecanolysis in interleukin-1 treated bovine cartilage Osteoarthritis Cartilage 2005 13 269 277 10.1016/j.joca.2004.10.023 15780640 PMC2771540

[B26] Karsdal MA Madsen SH Christiansen C Henriksen K Fosang AJ Sondergaard BC Cartilage degradation is fully reversible in the presence of aggrecanase but not matrix metalloproteinase activity Arthritis Res Ther 2008 10 R63 10.1186/ar2434 18513402 PMC2483454

[B27] Sawaji Y Hynes J Vincent T Saklatvala J Fibroblast growth factor 2 inhibits induction of aggrecanase activity in human articular cartilage Arthritis Rheum 2008 58 3498 3509 10.1002/art.24025 18975307

[B28] Gurley KA Reimer RJ Kingsley DM Biochemical and genetic analysis of ANK in arthritis and bone disease Am J Hum Genet 2006 79 1017 1029 10.1086/509881 17186460 PMC1698704

[B29] Hoyland JA Le Maitre CA Freemont AJ Investigation of the role of IL-1 and TNF in matrix degradation in the intervertebral disc Rheumatology 2008 47 809 814 10.1093/rheumatology/ken056 18397957

[B30] Park JB Park IC Park SJ Jin HO Lee JK KD R Anti-apoptotic effects of caspase inhibitors on rat intervertebral discs J Bone Joint Surg Am 2006 88 771 779 16595467 10.2106/JBJS.E.00762

[B31] Burke JG Watson RWG McCormack D Dowling FE Walsh MG JM F Intervertebral discs which cause low back pain secrete high levels of proinflammatory mediators J Bone Joint Surg Br 2002 84 196 201 10.1302/0301-620X.84B2.12511 11924650

[B32] Kang JD Stefanovic-Racic M Mcintyre LA Georgescu H Evans H Toward a biochemical understanding of human intervertebral disc degeneration and herniation. Contributions of nitric oxide, interleukins, prostaglandin E2, and matrix metalloproteinases Spine 1997 22 1065 1073 10.1097/00007632-199705150-00003 9160463

[B33] Studer RK Gilbertson LG Georgescu H Sowa G Vo N Kang JD p38MAPK inhibition modulates rabbit nucleus pulposus cell response to Il-1 J Orthop Res 2008 26 991 998 10.1002/jor.20604 18302237

[B34] Donohue PJ Jahnke MR Blaha JD Caterson B Characterization of link protein(s) from human intervertebral disc tissues Bioch J 1988 251 739 747 10.1042/bj2510739 PMC1149066 3415643

[B35] Pearce RH Mathieson JM Mort JS Roughley P Effect of age on the abundance and fragmentation of link protein of the human intervertebral disc J Orthop Res 1989 7 861 867 10.1002/jor.1100070612 2795326

[B36] Kim HJ Minashima T McCarthy EF Winkles JA Kirsch T Progressive ankylosis protein [ANK] in osteoblasts and osteoclasts control bone formation and bone remodeling J Bone Miner Res 2010 25 1771 1783 10.1002/jbmr.60 20200976 PMC3153348

[B37] Schimmer AD Dalili S Batey RA Riedl SJ Targeting XIAP for the treatment of malignancy Cell Death Differ 2006 13 179 188 10.1038/sj.cdd.4401826 16322751

