# Supplementary material for: Notochordal cells protect nucleus pulposus cells from degradation and apoptosis: implications for the mechanisms of intervertebral disc degeneration
Source: Arthritis Res Ther. 2011 Dec 29;13(6):R215. doi: 10.1186/ar3548 (PMC3334668; doi:10.1186/ar3548)
Supplement: Additional file 1 — Figure S1: Activated caspase-3 activity in NP cells treated with IL-1β + FasL for 24 and 48 hours (expressed relative to untreated cells). There is a clear and significant induction of cleaved/activated caspase-3 in the 48-hour treatment group verifying the induction of apoptosis. This is the same trend observed with fluorescent activated cell sorting analysis using Annexin V and Propidium Iodide. (Y axis refers to luminescence units/hour). [file ar3548-S1.DOC]

Supplementary Data Figures

Activated Caspase-3 Optimization:

Figure S1: Activated Caspase-3 activity in NP cells treated with IL-1β+FasL for 24 and 48 hours (expressed relative to untreated cells). There is a clear and significant induction of cleaved/activated Caspase-3 in the 48-hour treatment group verifying the induction of apoptosis. This is the same trend observed with fluorescent activated cell sorting analysis using Annexin V and Propidium Iodide. (Y axis refers to luminescence units/hour).
